# Parasitic plants have increased rates of molecular evolution across all three genomes

**DOI:** 10.1186/1471-2148-13-126

**Published:** 2013-06-19

**Authors:** Lindell Bromham, Peter F Cowman, Robert Lanfear

**Affiliations:** 1 Centre for Macroevolution and Macroecology, Research School of Biology, Australian National University, Canberra, A.C.T, 0200, Australia

**Keywords:** Mutation, Substitution, Comparative, Population size, Molecular rates

## Abstract

**Background:**

Theoretical models and experimental evidence suggest that rates of molecular evolution could be raised in parasitic organisms compared to non-parasitic taxa. Parasitic plants provide an ideal test for these predictions, as there are at least a dozen independent origins of the parasitic lifestyle in angiosperms. Studies of a number of parasitic plant lineages have suggested faster rates of molecular evolution, but the results of some studies have been mixed. Comparative analysis of all parasitic plant lineages, including sequences from all three genomes, is needed to examine the generality of the relationship between rates of molecular evolution and parasitism in plants.

**Results:**

We analysed DNA sequence data from the mitochondrial, nuclear and chloroplast genomes for 12 independent evolutionary origins of parasitism in angiosperms. We demonstrated that parasitic lineages have a faster rate of molecular evolution than their non-parasitic relatives in sequences for all three genomes, for both synonymous and nonsynonymous substitutions.

**Conclusions:**

Our results prove that raised rates of molecular evolution are a general feature of parasitic plants, not confined to a few taxa or specific genes. We discuss possible causes for this relationship, including increased positive selection associated with host-parasite arms races, relaxed selection, reduced population size or repeated bottlenecks, increased mutation rates, and indirect causal links with generation time and body size. We find no evidence that faster rates are due to smaller effective populations sizes or changes in selection pressure. Instead, our results suggest that parasitic plants have a higher mutation rate than their close non-parasitic relatives. This may be due to a direct connection, where some aspect of the parasitic lifestyle drives the evolution of raised mutation rates. Alternatively, this pattern may be driven by an indirect connection between rates and parasitism: for example, parasitic plants tend to be smaller than their non-parasitic relatives, which may result in more cell generations per year, thus a higher rate of mutations arising from DNA copy errors per unit time. Demonstration that adoption of a parasitic lifestyle influences the rate of genomic evolution is relevant to attempts to infer molecular phylogenies of parasitic plants and to estimate their evolutionary divergence times using sequence data.

## Background

Theoretical models have led to the prediction that parasites should evolve higher mutation rates in order to out-evolve their hosts [1]. This prediction has received support from experimental populations of bacteria [2,3]. But does it also apply to multicellular eukaryotes? A number of molecular phylogenetic studies have noted that parasitic taxa have much longer branch lengths, suggesting elevated rates of molecular evolution [4-7]. While it would not be surprising to find that specific genes involved in host-parasite interaction experience elevated substitution rates because of specific selection pressures [8], these studies report higher substitution rates in “housekeeping” genes that are not specifically connected to host-parasite interaction. It is therefore pertinent to ask whether being a parasite causes an increase in the genome-wide rate of molecular evolution in multicellular organisms.

Parasitic plants represent an ideal case for testing the hypothesis that parasites have faster rates of molecular evolution. They show a range of adaptations to parasitism and vary in the type and degree of interaction with their hosts [9,10]. Non-photosynthetic holoparasites are completely dependent on their host for energy, whereas hemiparasites retain photosynthetic ability. Hemiparasites can be either obligate (host-dependent) or facultative (able to be free-living). Parasitic plants also differ in their physical connection to their host (e.g., through the stems or roots), and the degree of reduction of their own anatomy (e.g., loss of stems, leaves or roots).

Faster substitution rates have been reported for a number of genes in several parasitic plant lineages [6,7,11-14]. Furthermore, the mode of parasitism has been suggested to influence rates of molecular evolution, for example holoparasites have been shown to have faster rates than confamilial hemiparasites in three plastid genes of the diverse broomrape family Orobanchaceae [4]. However, not all studies have demonstrated a general increase in substitution rate in parasitic plants. For example, a study of substitution rates in the 18S rRNA gene found that less than half of the parasitic taxa had elevated rates of molecular evolution (3 out of 16 hemiparasitic taxa, and 8 out of 17 holoparasites [7]), and only some of the nuclear genes in a study of substitution rates in the holoparasitic root parasite *Balanophora* showed significantly increased rates [13]. So the generality and possible causes of the increase in rates in parasite genomes remain unknown.

There are several reasons why a wide-ranging comparative study of rates of molecular evolution in parasitic plants is needed. Most importantly, a comparative study allows us to move from anecdotal observation to general principle. Although faster rates of molecular evolution have been noted in several different species of parasitic plants, we cannot tell from those studies alone whether accelerated rates are a general consequence of their parasitic lifestyle, or whether the faster rates in these particular species and genes are incidental to parasitism. By examining rate variation for all available parasitic lineages in the same analysis we can test the generality of the pattern.

A phylogenetic comparative study also provides a statistically rigorous way to test the hypothesis that parasitic lifestyle increases the rate of molecular evolution. Previously published studies have used a range of data, different methods, and different statistical approaches. In particular, many have used overlapping comparisons between parasites and non-parasites [6,13,15], or treated each parasitic lineage as an independent data point [7], violating assumptions of statistical independence inherent in the tests [16]. It is therefore important to analyse all of the available data within a single statistical framework to describe general patterns in molecular evolution in all independent lineages of parasitic plants.

A comparative study also allows us to compare all three plant genomes, to check for consistency of patterns of molecular evolution. For example, we might expect that the chloroplast genome would evolve rapidly if released from selective constraint, but it is not clear that the same explanation would apply to genes from the mitochondrial or nuclear genomes [7]. Studies that have reported higher rates in parasitic plants have not tested different possible explanations of this pattern, such as positive selection, relaxed purifying selection, or changes in population size. By comparing different substitutions rates within the same analytical framework, we can examine the reasons for the proposed acceleration in rates in parasitic plant taxa, by teasing apart the patterns due to mutation rate, selection and population processes.

There are many independent origins of parasitic lifestyle in angiosperms [17], so we are able to analyse data from 12 separate lineages of parasites. These lineages vary widely in growth form, life cycle, distribution and level of specialisation to parasitism. We are able to combine sequence data from chloroplast, nuclear, and mitochondrial sequences for representative taxa from all 12 lineages with data from their nonparasitic relatives. This provides sufficient data to allow the relationship between parasitism and faster rates to be examined within a single phylogenetic comparative framework.

## Results

**Figure 1:**
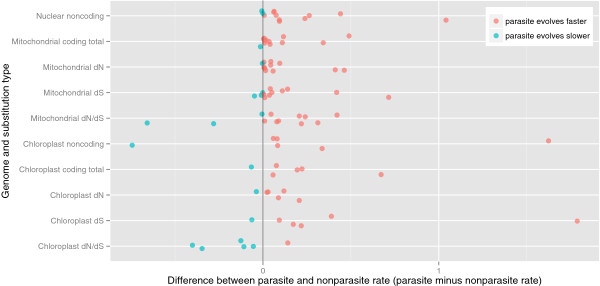
**Scatter plot of comparisons between phylogenetically independent pairs of parasitic plants and their nonparasitic relatives.** Points were calculated as the value of the nonparasitic clade subtracted from the parasitic clade, so that if the parasite has a faster rate then the difference is greater than 0. Each line represents a single substitution class in one of the three plant genomes, and each comparison is represented either by a red dot if the parasitic clade has the greater substitution rate or a blue dot if the nonparasitic clade has the higher substitution rate. The points have been slightly jittered to allow overlapping points to be seen clearly.

Parasitic plant clades had significantly higher substitution rates in all measures of branch length for all three genomes (Figure 1). Ten out of twelve nuclear comparisons showed a faster substitution rate in the parasitic lineage compared to its nonparasitic relative (Wilcoxon signed ranks test: Z = 2.786, p = 0.005). For the mitochondrial genes, all but one comparison had longer branch lengths in the parasitic clade (Z = 2.628, p = 0.009) and both nonsynonymous (dN: Z = 2.942, p = 0.003) and synonymous rates (Z = 2.157, p = 0.031) were significantly higher in the parasitic lineages.

For the chloroplast sequences, there were fewer sequences available, and neither the rRNA nor the protein-coding sequences had significantly different rates in parasites under the Wilcoxon signed ranks test. However, there was a clear trend in the data. For those comparisons with 16S rRNA (*rrn16*), seven out of eight comparisons has faster rates in the parasitic lineage. For the chloroplast protein-coding alignment, five out of six comparisons had faster rates in the parasitic lineage. We tested for a significant pattern across all comparisons by asking whether the parasite had the faster rate in whichever chloroplast genes were available, considering only the sign but not the magnitude of the branch length difference. Ten out of twelve comparisons had faster rates in the parasitic clade in the chloroplast sequences available for that comparison (two tailed sign test, p = 0.039). Because there are only 6 comparisons with chloroplast protein coding genes, it is difficult to draw any conclusions regarding synonymous and nonsynonymous substitution rates, but they do follow the same pattern as the mitochondrial and nuclear genomes, with both raised dS and dN in most parasite lineages, but not increased dN/dS.

We did not find a consistent difference in GC content between sequences from parasitic and non-parasitic taxa for nuclear (signs test: p = 0.77), mitochondrial (p = 0.15) or chloroplast (p = 0.15) alignments. This suggests that GC bias is unlikely to explain the consistent differences in rate between parasites and their non-parasitic relatives.

## Discussion

Our analysis provides strong support for the hypothesis that parasitic plants have consistently faster rates of molecular evolution than their non-parasitic relatives in mitochondrial, chloroplast, and nuclear sequences. The pattern is detected in all classes of substitutions tested, including ribosomal rRNA genes, and both synonymous and nonsynonymous substitutions in protein coding genes.

What could increase the rate of substitution so consistently in such varied lineages of parasitic plants? The parasitic plants included in this study range from fungus-like holoparasites, such as the achlorophyllous members of the Apodanthaceae that exist entirely within host tissues except when flowering, to perennial shrubs and trees, such as the sandalwoods (e.g. *Nestronia*) [18]. Here, we will consider several possible explanations for faster substitution rates in parasites.

### Relaxed selection

By drawing on metabolites from another individual, a parasite may be under less pressure to maintain their own means to produce those resources. Indeed, there may be selection pressure to lose unnecessary features, in order to save resources or accelerate the life cycle. Many holoparasites have lost or greatly reduced their leaves and stems, and in extreme cases the main vegetative body of the parasitic plant consists of roots growing as a mycelium-like mass inside the host tissues (e.g. Rafflesiaceae, Cytinaceae, Apodanthaceae). In such circumstances we would expect the genes that make products and structures needed for photosynthesis, such as leaves and chlorophyll, to decay into pseudogenes or be lost from the genome [19-22].

A gene that has no selective pressure to maintain function should acquire substitutions at a rate determined by the mutation rate. In this case, we would expect the nonsynonymous substitution rate to approximate the synonymous rate, and their ratio to be approximately 1. We do not find any evidence to support this effect in our study, where values of the ratio of dN to dS are not consistently closer to one in the parasitic lineages, even in the available protein-coding genes of the chloroplast [14]. It is possible that dN/dS values less than one in the parasitic lineage reflect past (negative) selection. Since our substitution rates are measured from the common ancestor of the sister clades, the genes in the parasitic clade will have been in autotrophic plants for some part of their history. In this case, we might expect dN/dS ratios to be higher (closer to one) in most parasitic plants when compared to their non-parasitic relatives, as they would have accumulated relatively more nonsynonymous substitutions during the parasitic part of their evolutionary history. But there is no clear trend toward higher dN/dS in parasitic plant lineages in any of the sequences: parasitic lineages have higher dN/dS in 9 out of 12 comparisons for mitochondrial genes (Z = 1.294, p = 0.196), and only 1 out of 6 comparisons for chloroplast genes (Z = −1.26, p = 0.21).

Loss of function in photosynthesis genes might not follow a simple decay from the adoption of parasitic lifestyle onwards. Chloroplast genes in different species of *Orobanche* (broomrape), a non-photosynthetic root parasite, show variable rates of decay into pseudogenes [23], and parasitic plants show different degrees of loss of genes from the plastid genome [20]. Some parasitic plants retain low levels of photosynthetic activity, insufficient to live independently, but enough to tide them over while waiting to find a suitable host [24]. Adaptation to parasitism may come about through changes to gene expression levels, leaving less of a signature in the coding sequences of genes [25].

Chloroplast genes in parasites are not necessarily divorced from the effects of selection. Chloroplasts genomes are not solely directed toward photosynthesis, they also contain genes that play a variety of key metabolic roles [19,26]. In any case, it is difficult to imagine that all the genes in this study are under relaxed purifying selection in parasites. The nuclear genes analysed code for essential parts of the protein synthesis machinery, and the mitochondrial genes are associated with gene expression and metabolism. Furthermore, we would not expect relaxation of selection to have a marked effect on synonymous substitution rates. So it does not seem very likely that the acceleration in both synonymous and nonsynonymous substitution rates across all three genomes in a wide range of parasitic plants can be explained through general relaxed selection on gene function.

### Positive selection

Hosts may use a number of different strategies to resist parasitic plants, operating at all stages of parasite establishment, ranging from immune-like responses, expression of parasite-specific toxins, and silencing host genes that make products essential for parasite growth [27]. So parasites will probably have to battle their hosts on multiple fronts, suggesting that many parts of parasite physiology and development, and consequently many different genes, will be under the strong selective pressure of a host-parasite arms-race. Indeed, transcriptomics of tissues at the host-parasite interface suggest that many pathways upregulated in the parasites are “general purpose” genes involved in core processes in both parasite and host species [28]. So selection could raise the substitution rate in a wide variety of genes involved in successful exploitation of hosts, including genes that might be considered “house keeping genes” [5]. The arms race between host and parasite may account for the divergence of parasitic lineages onto different host plant populations, such that parasites can be more successfully grown on their local host plants than those from another population [23].

Positive selection on protein-coding genes could raise the non-synonymous substitution rate, as it could drive the fixation of mutations that altered the functional properties of the protein. It is possible, therefore, that positive selection could explain part of the elevation of non-synonymous substitution rates we observed in parasitic plant lineages. However, we would not expect the synonymous substitution rate to be elevated by a host-parasite arms race of this kind. While some synonymous mutations could experience a degree of selective pressure [29], for example for translational efficiency [30,31], it is difficult to see how this could differ sufficiently between parasitic and autotrophic plants to explain the acceleration in synonymous substitution rates in parasites seen in this study. So if positive selection had raised the non-synonymous substitution rate but left the synonymous substitution rate relatively unchanged in parasitic lineages, then we would expect these lineages to show increased dN/dS relative to non-parasitic lineages. We observe no consistent changes in dN/dS associated with parasitic lineages in our data, suggesting that positive selection is unlikely to explain any significant proportion of the elevated parasitic substitution rates that we have observed.

### Reduction in effective population size

Effective population size (N_e_) affects patterns and rates of substitution, so should always be considered when seeking an explanation for consistent patterns in molecular evolution [32-34]. Parasitic taxa might undergo more frequent and severe founder effects, which might decrease their long-term effective population size and so increase the rate of substitution [5,35]. Endosymbiotic bacteria and fungi have higher substitution rates than their free-living relatives, which has been interpreted as a result of consistently lower population sizes and frequent population bottlenecks [36]. Could small population size or frequent population bottlenecks explain the faster rates in parasitic plants?

It is not clear that parasitic plants will typically have significantly smaller effective population sizes than their autotrophic relatives. Many are animal pollinated, indeed the flowers may be the most conspicuous part of the life cycle, like the gigantic fly-pollinated *Rafflesia* flowers, and many species have edible seeds that are likely to be dispersed by mammals or birds [18]. Some parasitic plants are widespread generalists that can tolerate a range of conditions and grow on a variety of host plants, to the extent that they are economically important weeds of many different crops [37]. So there seems no *a priori* reason to suspect that parasitic plants are less likely to be outcrossed or widely dispersed compared to their autotrophic relatives. In any case, lower average population sizes in parasitic lineages should mimic the effects of relaxed purifying selection, on a genome-wide scale, because selection becomes less efficient at removing slightly deleterious mutations in smaller populations [38]. We don’t detect any evidence of this in our study, because there is no significant increase in the ratio of nonsynonymous to synonymous substitutions in the parasitic taxa.

### Increased mutation rate

Parasites will often have much faster rates of molecular evolution than their hosts, potentially due to their much faster rate of generation turnover [39], which may help them get ahead in the arms race [40]. But in this study, we have not compared the rate of molecular evolution of parasites to their hosts, but of parasites to their free-living relatives. Synonymous substitution rate (dS) is commonly interpreted as reflecting the mutation rate, because changes to the gene sequence that do not cause a change in the protein-product are likely to be invisible to selection [41]. However, other factors could influence the synonymous substitution rate, such as GC biased gene conversion [42] and codon-usage bias [29], and synonynomous substitution rate can vary across the genome [29,43]. But while dS is not a perfect predictor of mutation rate, changes in mutation rate should be reflected in changes in the rate of both synonymous and nonsynonymous substitution. So elevated mutation rates in parasites could produce the pattern we observe in this study, raising both the synonymous and non-synonymous substitution rates, but not influencing the dN/dS ratio. If elevated mutation rates can explain the patterns we have observed, we must explain how adopting a parasitic lifestyle could result in an increase in the mutation rate.

Simple models that set up an arms race between host and parasite loci predict that increased mutation rate will allow parasites to “chase” the host’s changing defences [1]. Mutator alleles can speed evolution in a clonally reproducing population even when they are not fixed in the population [44]. However, the increase in mutation predicted by chase models is typically transient [45], as the positively selected mutations recombine away from mutator alleles. So alleles that cause a global increase in mutation rate are unlikely to be maintained by selection for the novel traits they generate [46]. The link between mutation rate and adaptive evolution is complex even in asexual lineages [47,48], and in sexual populations, it is not clear the extent to which adaptation will be mutation limited [49]. It seems unlikely that a sexually reproducing parasitic plant would gain enough advantage from increased mutation rate, in terms of evading host defence, for selection on mutator alleles to outweigh the costs of producing more deleterious mutations.

Alternatively, increased mutation rate in parasites might results from an erosion of the DNA repair systems that maintain replication fidelity and fix incidental damage. For example, it has been suggested that the lower bound of the mutation rate is set by the efficiency of selection for maintenance or improvement to DNA repair, which will be primarily limited by the effective population size [50]. In this case, we might expect to see that dS correlates with N_e_, so if parasitic plants had consistently lower population sizes it may increase dS and dN, potentially accounting for the lack of correlation between parasitism and dN/dS. However, it is difficult to assess this explanation without an independent way of estimating N_e_ for these species.

The two explanations just discussed both concern the role of selection in shaping mutation rates, whether increased positive selection for novelty or less efficient negative selection on DNA copy fidelity and repair. In addition, there are many other species traits that can influence mutation rates, so if these traits differ consistently between parasites and their nonparasitic relatives, it could potentially explain the pattern in rates of molecular evolution. The most obvious candidate is number of DNA replications per unit time.

Species with shorter generation times tend to have higher rates of molecular evolution, presumably because they accumulate more DNA replication errors per unit time [51]. In plants, it has been widely reported that annual plants and herbs have faster substitution rates than perennials and woody plants, respectively [52], though it is not clear exactly how this relates to number of genome copies per unit time [29]. Plant height also correlates with rate of molecular evolution in plants, which may be because taller plants tend to have longer generation times (and so fewer meiosis per unit of time) and slower average growth rates (and so fewer mitoses per unit of time) [53]. Highly reduced holoparasites that do not produce stems, such as *Pilostyles* and *Apodanthes,* are clearly much shorter than their freeliving relatives, such as *Hibiscus* and *Theobroma* (Additional file 1: Table S1). Similarly, the fungus-like *Cynomorium coccineum* is much smaller than its nonparasitic relative *Peridiscus lucidus*, a South American tree species. So if parasitic plants tend to have smaller bodies, shorter lives or more rapid generation turnover than their nonparasitic relatives, then they might also have higher mutation rates because their genomes will undergo more replications per unit time.

Although we do not have reliable measures of average height of all species in our comparisons, we can make a broad assessment based on growth form. In the majority of comparisons, the parasitic species are clearly smaller than the nonparasitic species – specifically in the Apodanthaceae (comparison 1), Cytinaceae (2), Rafflesiaceae (3), Cynomoriaceae (4), Mitrastemonaceae (6), Lauraceae (10), Hydnoraceae (11), and the clade consisting of the parasitic relatives of the Olacaceae (12). These comparisons all show higher rates in the parasitic species for the nuclear genes and mitochondrial genes except for comparison 6 (Mitrastemonaceae) which has slower rates for the parasitic species for mitochondrial dS and chloroplast 16S rRNA.

While it provides a plausible explanation of the patterns we observe, the plant height hypothesis does not provide a perfect fit to all of the substitution rate differences in this study. For example, the perennial hemiparasite *Krameria lanceolata* has lower nuclear rates and mitochondrial dS than the annual herb *Kallstroemia parviflora*, which is consistent with larger size in the parasitic taxon, but it also has faster mitochondrial dN. For the chloroplast comparison, *Krameria* has lower substitution rates than its larger relatives, the tree *Guaiacum sanctum* and the woody shrub *Larrea cuneifolia.* For the remaining two comparisons (9 and 10), there is not a clear difference in body size, but the parasites have the higher substitution rates in all alignments. So while the patterns we observe are broadly consistent with an effect of plant size on rates of mutation rates, the match to the observations is not exact, and there may be other factors at play.

Another potential source of an indirect link between molecular evolution and parasitic lifestyle is through environmental effects. It has been suggested that plant lineages at low latitudes have increased rates of molecular evolution, potentially due to the mutagenic effect of UV radiation, or to higher growth rates and faster generation turnover [53-55]. But latitude or environmental energy is unlikely to provide an explanation for higher substitution rates in parasitic plants. Some parasitic plant groups are primarily tropical, such as the Rafflesiaceae, found in South East Asia. But most of the parasitic lineages included in this study have a broader distribution, including both temperate and tropical species, for example the Orobanchaceae (broom rapes) and *Cuscuta* (dodder). Similarly, net diversification rate in plants has been linked to both synonymous and nonsynonymous rates [56,57], but since parasitic plant clades tend to be less diverse than their nonparasitic sister clades [58], this seems unlikely to provide an explanation for our results.

## Conclusions

The study of parasitic plants has many important outcomes. By reducing the water and nutrients available to host plant growth, parasitic plants have a serious impact on agriculture, which is estimated to impact on the food supply of over 100 million people [27]. Parasitic plants also provide an excellent case study for examining many evolutionary processes, including diversification and specialization, genome evolution, and host-parasite interactions. Demonstrating consistently higher substitution rates in parasitic plants not only provides a window on molecular evolution, it also has practical implications for the use of DNA sequence data in evolutionary and ecological research. Molecular markers have played a key role not only in establishing the phylogeny and systematics of parasitic plants, but in understanding broader evolutionary patterns, such specialization to different host plants. Consistent differences in rate of molecular evolution in parasitic lineages should be considered when interpreting molecular phylogenies and date estimates.

## Methods

### Data

We gathered DNA sequence data from GenBank for each of the parasitic plant clades identified by Barkman *et al*. [17]. We focus on angiosperms, so we do not include the gymnosperm *Parasitaxus,* nor the liverwort *Aneura mirabilis,* both of which appear to form parasitic attachments to host plants via a fungal partner [20,59]. Nor do we include mycoheterotrophic angiosperm species, such as those in the Ericaceae [60], which derive part of their nutrition through a relationship with fungi, because the line between fully parasitic mycoheterotrophs and plants that have mycorrhizal partners is not always clear [61,62].

We use a sister-clades approach, identifying clades of parasitic plants and their autotrophic (non-parasitic) relatives. Each lineage of the sister clade has had the same amount of time since their last common ancestor to accumulate substitutions, therefore any difference in branch length (estimated number of substitutions since the last common ancestor) is likely to reflect a difference in the net substitution rate [16,56]. If parasitic plants have a higher rate of molecular evolution, then we would expect to see a longer total branch length in the parasitic clade compared to its non-parasitic sister clade [51].

The position of parasitic clades within angiosperm phylogeny has been controversial, and even the higher-level relationships are debated [63-65]. As a consequence, there have been many changes to higher-level taxonomy. For simplicity, we report the GenBank taxonomy specified for each sequence used.

We identified close non-parasitic relatives of each of the parasitic lineages, using published phylogenies and taxonomies as a guide [17,64-72]. For the purposes of this study, we do not need to know the exact phylogenetic position of parasitic taxa, as long as we can be sure that we are comparing phylogenetically independent origins of parasitic lineages to their autotrophic relatives. For each independently evolved parasitic lineage we aimed to select a related autotrophic clade that could be placed outside the parasitic clade with certainty, so we did not always choose the closest relative where phylogeny was uncertain. This gave us 12 phylogenetically independent comparisons between parasitic and non-parasitic lineages (Tables 1 and 2). Each parasitic and non-parasitic sister clade was represented by sequences from up to nine different species (see Additional file 1: Table S1, Additional file 2: Table S2 and Additional file 3: Table S3). We always balanced the number of species in the sister clades, to minimize the effects of node density on substitution rate estimation [16].

**Table 1:** Estimates of branch length for sister clades of parasitic (P) and autotrophic (non-parasitic, NP) plants for nuclear and mitochondrial sequences

**Comparison**	**Parasitic (P)**	**Non-parasite (NP)**	**Nuclear**			**Mitchondrial (all substitutions)**			**Mitchondrial (dN)**			**Mitchondrial (dS)**			**Mitchondrial (dN/dS)**		
			**P**	**NP**	**Sign**	**P**	**NP**	**Sign**	**P**	**NP**	**Sign**	**P**	**NP**	**Sign**	**P**	**NP**	**Sign**
1	Apodanthaceae	Malvaceae	0.513	0.072	+	0.380	0.037	+	0.466	0.055	+	0.739	0.024	+	0.628	1.287	–
2	Cytinaceae	Thymelaeaceae	0.121	0.028	+	0.070	0.035	+	0.077	0.032	+	0.094	0.043	+	0.960	0.742	+
3	Rafflesiaceae	Passifloraceae/ Euphorbiaceae	1.093	0.052	+	0.631	0.141	+	0.577	0.114	+	0.660	0.241	+	0.726	0.520	+
4	Cynomoriaceae	Hamamelidacea/ Peridiscaceae	0.087	0.012	+	0.030	0.011	+	0.017	0.007	+	0.060	0.024	+	0.276	0.282	–
5	Krameriaceae	Zygophyllaceae	0.019	0.025	–	0.015	0.011	+	0.012	0.007	+	0.023	0.025	–	0.529	0.289	+
6	Mitrastemonaceae	Vaccinieae	0.075	0.013	+	0.026	0.017	+	0.021	0.005	+	0.039	0.048	–	0.529	0.108	+
7	Boraginaceae	Boraginaceae	0.036	0.028	+	0.029	0.020	+	0.020	0.013	+	0.049	0.044	+	0.389	0.343	+
8	Orobanchaceae/ Rhinantheae	Lamiliales/ Plantaginaceae	0.042	0.043	–	0.007	0.022	–	0.002	0.006	–	0.023	0.072	–	0.133	0.054	+
9	Convolvulaceae	Ipomoeeae	0.080	0.016	+	0.061	0.021	+	0.052	0.008	+	0.096	0.054	+	0.562	0.249	+
10	Lauraceae	Lauraceae	0.323	0.230	+	0.012	0.005	+	0.010	0.004	+	0.018	0.008	+	0.540	0.530	+
11	Hydnoraceae	Aristolochiaceae	0.296	0.057	+	0.152	0.042	+	0.072	0.014	+	0.152	0.042	+	0.418	0.330	+
12	Balanophoraceae/Loranthceae/ Schoepfiaceae/ Olacaceae	Olacaceae	0.384	0.120	+	0.131	0.014	+	0.101	0.005	+	0.180	0.040	+	0.493	0.773	–
**Wilcoxon signed-ranks test**			**Z = 2.786, p = 0.005**			**Z = 2.628, p = 0.009**			**Z = 2.942, p = 0.003**			**Z = 2.157, p = 0.031**			**Z = 1.294, p = 0.196**		

For details of the species and sequences included in each comparisons see Additional file 1: Table S1 and Additional file 2: Table S2. Mitochondrial branch lengths were estimated for the whole alignment (all substitutions), as well as nonsynonymous (dN) and synonymous substitutions (dS): see methods for details. Sign indicates whether the parasite has the longer branch length (+) or not (−). The Wilcoxon ranked signs test takes into account the magnitude of differences in branch length as well as the sign.

**Table 2:** Estimates of branch length for sister clades of parasitic (P) and autotrophic (non-parasitic, NP) plants for chloroplast sequences

**Comparison**	**Parasitic clade**	**Non-parasite**	**16S rRNA ( *rrn16 *)**			**Protein-coding (all)**			**Comb.**	**dN**			**dS**			**dN/dS**		
			**P**	**NP**	**Sign**	**P**	**NP**	**Sign**	**Sign**	**P**	**NP**	**Sign**	**P**	**NP**	**Sign**	**P**	**NP**	**Sign**
1	Apodanthaceae	Malvaceae	1.637	0.013	+				+									
2	Cytinaceae	Thymelaeaceae	0.091	0.008	+				+									
3	Rafflesiaceae	Passifloraceae/ Euphorbiaceae	0.150	0.091	+				+									
4	Cynomoriaceae	Hamamelidacea/ Peridiscaceae	0.083	0.002	+				+									
5	Krameriaceae	Zygophyllaceae				0.098	0.164	–	–	0.032	0.070	–	0.296	0.360	–	0.112	0.220	–
6	Mitrastemonaceae	Vaccinieae	0.196	0.940	–				–									
7	Boraginaceae	Boraginaceae				0.076	0.019	+	+	0.040	0.017	+	0.133	0.040	+	0.297	0.423	–
8	Orobanchaceae	Plantaginaceae	0.027	0.001	+	0.561	0.366	+	+	0.413	0.206	+	0.960	0.743	+	0.409	0.268	+
9	Convolvulaceae	Convolvulaceae/ Ipomoeeae	0.014	0.001	+	0.724	0.052	+	+	0.115	0.027	+	1.938	0.151	+	0.047	0.102	–
10	Lauraceae	Lauraceae				0.344	0.122	+	+	0.235	0.116	+	0.526	0.137	+	0.320	0.721	–
11	Hydnoraceae	Aristolochiaceae	0.336	0.000	+				+									
12	Santalaceae/ Olacaceae	Strombosiaceae				0.125	0.049	+	+	0.068	0.039	+	0.241	0.069	+	0.239	0.585	–
**Wilcoxon rank sign test**			Z = 1.47, p = 0.14			Z = 1.68, p = 0.09				Z = 1.47, p = 0.14			Z = 1.89, p = 0.06			Z = −1.26, p = 0.21		
**Signs test**									**p = 0.039**									

For details of the species and sequences included in each comparisons see Additional file 3: Table S3. Protein-coding branch lengths were estimated for all substitutions (all), nonsynonymous (dN) and synonymous substitutions (dS): see methods for details. Sign indicates whether the parasite has the longer branch length (+) or not (−). The Wilcoxon ranked signs test takes into account the magnitude of differences in branch length as well as the sign. Because most comparisons had only 16S or the protein-coding genes, we combined the two (Comb.) to assess the pattern over all comparisons using a signs test: for this test we considered only the direction of the rate difference for each comparison, whether calculated from 16S or protein coding genes or both.

We targeted genes for which sequences were available for the largest number of the parasitic taxa and their non-parasitic relatives: maturase R (*matR*), cytochrome oxidase subunit I (*coxI*), NADH dehydrogenase subunit 1 (*nad1*) and ATPase F1 alpha subunit (*atp1*) from the mitochondrial genome; 18S small subunit ribosomal RNA (*rrn18*) and 26S ribosomal RNA (*rrn26*) from the nuclear genome; ribulose-1, 5-bisphosphate carboxylase/oxygenase large subunit (*rbcL*), maturase K (*matK*) and 16S ribosomal RNA (*rrn16*) from the chloroplast genome. We used the GeneFinder script [73] to find the longest available sequences for each gene on GenBank. Some gene sequences were unavailable for some taxa (in particular, chloroplast sequences were often not available for holoparasites). A complete list of the sequences, along with their GenBank accession numbers, is available in the Supplementary Material (Additional file 1: Table S1, Additional file 2: Table S2 and Additional file 3: Table S3).

### Analysis

We aligned sequences in Geneious version 6.0 [74]. We aligned protein-coding exons in frame, using the amino acid translations as a guide, with the MUSCLE translation alignment plugin [75], and we aligned non-coding sequences using the standard MUSCLE alignment plugin in Geneious. We then adjusted all alignments by eye. When we identified pseudogenes in chloroplast protein sequences we removed them from the alignments. We produced three separate genome alignments by concatenating the genes from each genome (mitochondrial, chloroplast, and nuclear). For each genome alignment, substitution models and partitioning schemes were chosen using AICc in Partition Finder [76]. The best substitution models and partition schemes were used for phylogenetic estimation in RAxML v7.0.4 [77]. Separate maximum likelihood analyses were run for each alignment.

Next we created a single maximum likelihood (ML) tree topology and corresponding alignment for each of the three genomes (nuclear, plastid, and mitochondrial) for each of the 12 sister pairs (36 topologies and alignments in total). For each comparison, we then extracted the parasitic and non-parasitic taxa plus a closely related non-parasite outgroup from a different order from each of the genome trees using the APE package [78], and produced corresponding alignment files. For each alignment, we only included gene regions that had coverage for both the parasitic and non-parasitic clades. We removed the small introns of the *coxI* and *nad1* genes in the mitochondrial alignment.

We estimated total branch length for all alignments using maximum likelihood and a GTR + G model in the baseml program of the PAML package [79]. For protein-coding genes (mitochondrial *matR*, *coxI*, *nad1* and *atp1;* chloroplast *rbcL* and *matK*), we estimated synonymous, and nonsynonymous branch lengths using the GY94 codon substitution model [80] in the codeml program of the PAML package [79], with dN/dS values free to vary across the tree. In addition, we estimated clade-specific dN/dS ratios (ω) for the parasitic and non-parasitic clade in each comparison.

To compare the rate of accumulation of substitutions in parasitic lineages to their nonparasitic sister lineage, we calculated the total branch length of the parasitic and non-parasitic clades in each sister pair using a python script. Total branch lengths for ribosomal RNA genes and protein-coding genes, and total dS and dN branch lengths for protein-coding genes were calculated by summing all edge lengths for each clade from the shared node with its sister clade. Since estimates of substitution rates can be influenced by base composition bias, we also calculated GC content for each sister clade. We tested for a significant association between parasitism and rate of molecular evolution using a Wilcoxon signed ranks test in the SPlus package v8.2.

## Availability of supporting data

Data has been deposited in the Data Dryad Repository: http://dx.doi.org/10.5061/dryad.fc74k.

## Competing interests

The authors declare that they have no competing interests.

## Authors’ contributions

All authors contributed to data collection and analysis and manuscript preparation and all authors read and approved the final manuscript.

## Supplementary Material

Additional file 1: Table S1 Nuclear comparisons. Details of comparisons between parasitic plants and their nonparasitic relatives. For each comparison (Comp.), we list the parasitic species and non-parasitic species included, with the GenBank accession numbers for sequences used in this analysis. We also list the parasitic mode for the parasitic species, whether Holoparasitic (Holo, for nonautotrophic species) or Hemiparasitic (Hemi, for species capable of photosynthesis). Note that some taxa include sequences from congeneric species. Click here for file

Additional file 2: Table S2 Mitochondrial comparisons. Details of comparisons between parasitic plants and their nonparasitic relatives. For each comparison (Comp.), we list the parasitic species and non-parasitic species included, with the GenBank accession numbers for sequences used in this analysis. We also list the parasitic mode for the parasitic species, whether Holoparasitic (Holo, for nonautotrophic species) or Hemiparasitic (Hemi, for species capable of photosynthesis). Note that some taxa include sequences from congeneric species. Click here for file

Additional file 3: Table S3 Chloroplast comparisons.Details of comparisons between parasitic plants and their nonparasitic relatives. For each comparison (Comp.), we list the parasitic species and non-parasitic species included, with the GenBank accession numbers for sequences used in this analysis. We also list the parasitic mode for the parasitic species, whether Holoparasitic (Holo, for nonautotrophic species) or Hemiparasitic (Hemi, for species capable of photosynthesis). Note that some taxa include sequences from congeneric species. Click here for file
